# International Medical Monographs

**Published:** 1913-09

**Authors:** 


**International Medical Monographs.** General Editors: Leonard Hill, M.B., F.R.S., William Bulloch, M.D. **Diabetes: its Pathological Physiology.** By John J. R. MacLeod, M.B., Ch.B., D.P.H. Pp. xi, 224. London: Edward Arnold. 1913. 10s. 6d. net.

In this work will be found a very able account of the experimental work by which the pathology of diabetes is gradually being elucidated. All clinical workers should have a knowledge of the extensive modern researches into this subject, as they form the scientific basis of accurate clinical diagnosis and treatment. This book can be confidently recommended to them as giving a clear and not too lengthy presentation of this difficult study. In spite of all the work that has been done, we are still far from understanding the essential nature of diabetes, and to no subject do the Hippocratic maxims that experiment is fallacious, true judgment difficult, more closely apply. Amongst the number of apparently contradictory experiments and rival theories the clinical student of diabetes may well feel bewildered, and in need of a trustworthy guide. He may turn with confidence for such critical guidance to this little book. We note that the views that sugar exists in the blood in a loose combination with proteid, and that glycolysis is dependent upon a pro-ferment furnished by the muscles and activated by an internal secretion of the pancreas, are held to be not proven. With regard to the action of the ductless glands, the author considers that present knowledge does not go further than to show that the adrenals, pancreas, parathyroids and possibly posterior lobe of pituitary are most important in the control of the carbohydrate metabolism, that their removal means loss of hormone control, and that in the case of the pancreas and parathyroid this hormone control facilitates and in that of adrenals depresses utilisation of sugar. The chapters on examination of urine, on the behaviour of sugar in the blood, and on the limits of assimilation will be found especially useful in clinical work. The bibliographies at the end of each chapter form a valuable feature of the work.

For much of our knowledge of the pathology of diabetes we are indebted to the researches of the author and his co-workers, and we cordially recommend his book as a most useful and timely one, and written with authority.

